# 30 YEARS OF BONE GIANT CELL TUMOR IN THE KNEE: A BRAZILIAN PERSPECTIVE

**DOI:** 10.1590/1413-785220253302e285061

**Published:** 2025-06-02

**Authors:** Eduardo da Silva Rodrigues, Michelle Ghert, Bruno Pereira Antunes, Carlos Roberto Galia, Julie Francine Cerutti Santos Pestilho, Eduardo Areas Toller, Olavo Pires de Camargo, Edgard Eduard Engel, Suely Akiko Nakagawa, Alex Guedes, Ricardo Gehrke Becker

**Affiliations:** 1 Hospital de Clínicas de Porto Alegre (HCPA), Porto Alegre, RS, Brazil.; 2 University of Maryland, College Park, MD, USA.; 3 Instituto do Câncer Infantil, Porto Alegre, RS, Brazil.; 4 Hospital de Amor, Barretos, SP, Brazil.; 5 Universidade de São Paulo, Hospital das Clínicas da Faculdade de Medicina, Instituto de Ortopedia e Traumatologia, Sao Paulo, SP, Brazil.; 6 Hospital das Clínicas de Ribeirão Preto, Ribeirao Preto, SP, Brazil.; 7 Hospital A. C. Camargo Câncer Center, Sao Paulo, SP, Brazil.; 8 Hospital Santa Izabel, Salvador, BA, Brazil.

**Keywords:** Neoplasms, Bone Tissue, Giant Cell Tumors, Curettage, Denosumab, Recurrence, Neoplasias de Tecido Ósseo, Tumor de Células Gigantes, Curetagem, Denosumabe, Recorrência

## Abstract

**Objective:**

To analyze the evolution of patient/tumor characteristics and treatments for GCTB in the knee in Brazil over 30 years and assess changes in local recurrence rates.

**Methods:**

Retrospective study of 335 patients (1989-2021) from 16 Brazilian centers. Data on patient/tumor characteristics, recurrence, metastasis, and treatment trends were evaluated.

**Results:**

Campanacci grade 3 tumors, pulmonary metastasis, and local recurrence rates were 56.7%, 5.3%, and 15.8%, respectively. Recurrence was 21.4% for curettage and 9% for resection. Curettage with denosumab showed 23.8% recurrence, versus 21% for curettage alone. Overall, local recurrence decreased from 22.9% (1989-2005) to 15.1% (2006-2021), with a significant drop after en bloc resection (23% to 7.8%), while curettage-related recurrence remained stable.

**Conclusions:**

Despite an increase in aggressive tumors, local recurrence decreased, especially after en bloc resection. These findings emphasize the challenges of managing rare diseases in emerging economies. ***Level of evidence: III; Retrospective Cohort Study.***

## RESUMO

**Objetivo:**

Analisar a evolução das características dos pacientes/tumores e dos tratamentos para GCTB no joelho no Brasil ao longo de 30 anos, avaliando as mudanças nas taxas de recorrência local.

**Métodos:**

Estudo retrospectivo de 335 pacientes (1989-2021) de 16 centros brasileiros. Foram avaliados dados sobre características dos pacientes/tumores, recorrência, metástases e tendências de tratamento.

**Resultados:**

As taxas de tumores Campanacci grau 3, metástase pulmonar e recorrência local foram de 56,7%, 5,3% e 15,8%, respectivamente. A recorrência foi de 21,4% para a curetagem e 9% para a ressecção. A associação de denosumabe e curetagem apresentou 23,8% de recorrência, contra 21% para a curetagem isolada. A recorrência local reduziu de 22,9% (1989-2005) para 15,1% (2006-2021), com uma queda significativa após a ressecção em bloco (23% para 7,8%), enquanto a recorrência após curetagem permaneceu estável.

**Conclusões:**

Apesar do aumento de tumores agressivos, a taxa de recorrência local diminuiu, especialmente após a ressecção em bloco. Esses achados destacam os desafios no manejo de doenças raras em economias emergentes. ***Nível de evidência: III; Estudo de Coorte Retrospectivo.***

## INTRODUCTION

Giant cell tumor of bone (GCTB) is a locally aggressive tumor predominantly presenting in the bones around the knee joint of adults between the ages of 20 and 50 years. GCTB represents 20% of benign bone tumors and up to 5% of all primary bone tumors, and the primary therapeutic approach is surgical, predominantly via curettage or *en bloc* resection. However, when there is an elevated risk of functional impairment, pain, or pulmonary involvement, alternative treatments, such as the RANK-L inhibitor Denosumab and bisphosphonates, may be considered. While curettage tends to yield better functional outcomes than resection, it is associated with a higher risk of local recurrence. Therefore, it is imperative to undertake a comprehensive risk stratification approach in order to ensure the selection of the most appropriate treatment and to minimize the probability of local recurrence.^1–4^

The knee's role as a crucial joint for movement and weight-bearing means that GCTB's presence can notably affect a patient's quality of life. The tumor's high recurrence risk post-curettage has been well-documented, with older studies indicating rates from 20 to 50%, although recent findings suggest a decline to closer to 11%.^5,6^ Such relapses can lead to severe complications, including loss of function, bone stock depletion, pathological fractures, and, on rare occasions, pulmonary metastasis, which is most prevalent post-recurrence, appearing in 2 to 5% of cases.^7^ Finally, given its proximity to essential structures, the management of GCTB in the knee remains a challenge, emphasizing the importance of appropriate and effective surgical management.^8^

In this study, we reviewed a large multicenter cohort of patients treated for GCTB of the knee in national tumor centers in Brazil over a 30-year period. The aim of the study was to assess patient and tumor characteristics and to describe the treatment outcomes of GCTB located around the knee in the context of an emerging economy in South America. We asked the following questions: (1) What are the patient/tumor characteristics of surgical cases of GCTB in the knee in Brazil over the past 30 years, and how have treatment methods, including surgical approaches, evolved during this period? (2) What was the rate of local recurrence, and did this change over time?

## METHODS

This study is a retrospective review of cases of GCTB of the knee identified in the databases of 16 specialized Brazilian institutions dedicated to the treatment of musculoskeletal tumors. Before the study began, we received ethical approval from the coordinating center and all participating institutions (REB# 94280918.0.0000.5327).

Outcome-related data were gathered from both electronic and paper medical records by all participating centers. To safeguard participant confidentiality, each individual was designated a numerical code. Data were transmitted to the coordinating center using an encrypted email system. Upon receipt, the data underwent meticulous examination to address any discrepancies or inconsistencies. Cases with conflicting variables were returned to the respective centers for clarification and then re-examined by the coordinating center. The collected data were stored in MS Excel and SPSS version 27.0 software programs.

Variables extracted were categorized into (1) demographic variables including gender, age, region within the country where the patient received treatment, and the timeframe of primary tumor diagnosis and treatment; (2) clinical variables at presentation including pulmonary metastasis, pathological fracture, and Campanacci grade^9^ based on radiographic appearance: Grade I (latent) characterized by well-defined borders, Grade 2 (active) with less distinct borders, and Grade 3 (aggressive) with a breached cortex and soft tissue extension; (3) treatment-related variables detailing the type of surgery (curettage, *en bloc*, amputation), type of filling (cement, bone graft, or none), surgical adjuvants used (single, combined, or none), and denosumab usage; and (4) the primary outcome of local recurrence. The sample was divided into two 15-year groups (1989 – 2005 and 2006 – 2021) based on the availability of magnetic resonance imaging (MRI) in Brazil. Advanced imaging modalities such as MRI have resulted in improved pre-operative planning for musculoskeletal neoplasms, potentially impacting local disease control.

**Figure 1:**
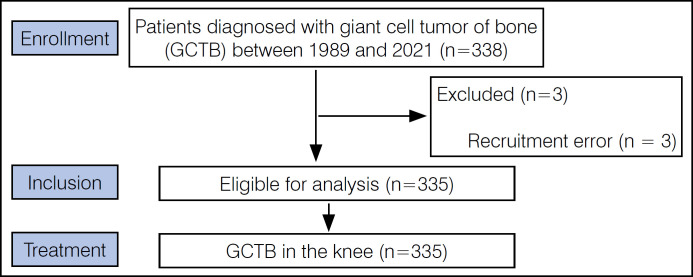
Flow diagram of patient selection.

The inclusion criteria were: (1) Pathological diagnosis of GCTB in the distal femur, proximal tibia, and proximal fibula; (2) Primary tumor treatment administered at one of the participating centers; (3) Availability of comprehensive medical records for analysis at the coordinating center. Fifty-five patients were excluded due to incomplete records. A total of 335 patients met the inclusion criteria (Figure 1). Collaborative efforts among participating entities identified and rectified data discrepancies and voids. Nonetheless, of the 335 patients evaluated, instances of missing information were noted in 4% regarding pulmonary metastases, 12% concerning pathological fractures, 0.3% on the type of cavity filling, and 0.9% related to denosumab utilization. These data deficiencies were predominantly due to the loss of historical medical records, as well as inconsistencies in documentation procedures across various participating institutions. The primary outcome examined was the rate of local recurrence and its change over time. Secondary outcomes analyzed included the rate of local recurrence based on the type of surgery, use of denosumab before curettage, number of adjuvants used prior to surgery, and tumor aggressiveness according to the Campanacci classification.

## RESULTS

### Patient and tumor characteristics

In this analysis of 335 patients with GCT of the knee, 183 (54.6%) were females, and 152 (45.4%) were males, with a median age of 33 years (range 14-74 years, standard deviation 12.6 years). The median follow-up duration was 89.6 months. Patients were mainly from the Southeast region of Brazil (55.5%), followed by the South (26.9%), Northeast (13.7%), and North (3.9%). The distal femur was the most affected site, comprising 52.2% of cases, followed by the proximal tibia (38.5%) and proximal fibula (9.2%). Notably, 56.7% of the tumors were classified as aggressive Campanacci grade 3. Pathological fractures were present in 16.7% of patients, mostly in the distal femur, and pulmonary metastases were detected in 5.3% at diagnosis. The recent cohort exhibited more aggressive tumors, evidenced by a rise in Campanacci grade 3 cases from 43% to 58%. Instances of pathological fractures remained relatively unchanged between the time periods (Table 1).

**Table 1:** Patients and tumor characteristics from 1989 to 2021.

Characteristics, %(n)	All patients	1989-2005	2006-2021
	(n=335)	(n=35; 10.4%)	(n=300; 89.6%)
Age (years)	33.0 (± 12.6)	29.7 (± 10.9)	33.4 (± 12.8)
**Sex**			
Male	45.4 (152)	37.1 (13)	46.3 (139)
Female	54.6 (183)	62.9 (22)	53.7 (161)
**Site of lesion**			
Distal femur	52.2 (175)	45.7 (16)	53.0 (159)
Proximal fibula	9.2 (31)	8.6 (3)	9.3 (28)
Proximal tibia	38.5 (129)	45.7 (16)	37.7 (113)
**Campanacci grade**			
1/2	43.3 (145)	57.1 (20)	41.7 (125)
3	56.7 (190)	42.9 (15)	58.3 (175)
**Regions**			
South	26.9 (90)	22.9 (8)	27.3 (82)
Northeast	13.7 (46)	0.0 (0)	15.3 (46)
Southeast	55.5 (186)	74.3 (26)	53.3 (160)
North	3.9 (13)	2.9 (1)	4.0 (12)
Pathological fracture	19 (56)	14.3 (5)	17.0 (51)
Pulmonary metastasis	5.3 (17)	2.9 (1)	5.3 (16)

Categorical variables are presented as frequencies and percentages, and continuous variables are presented as the mean and SD.

### Treatment characteristics

Surgical approaches were most commonly curettage (57%), followed by resection (38.5%), and amputation (3.9%). Among those undergoing curettage, 16.2% were managed without intraoperative adjuvant therapy, 40.8% with one adjuvant, and 42.9% with two or more adjuvants. In terms of cavity filling after curettage, cement filling was the most common (95.8%) approach, either alone or combined with bone graft, while bone graft alone (1.0%) and no filling (3.1%) were less common approaches. The surgical approaches were similar in the two time periods of 1989-2005 and 2006-2021. Curettage procedures comprised 60.0% and 57.0% in the respective time periods, with *en bloc* resections 37.1% and 38.9%. Amputation rates were also stable at 2.9% and 4.0% (Table 2).

**Table 2:** Treatment characteristics and surgical approach from 1989 to 2021.

Characteristics % (n)	All patients	1989-2005	2006-2021
	(n=335)	(n=35; 10.4%)	(n=300; 89.6%)
**Type of surgery**			
Curettage	57.0 (191)	60.0 (21)	57.0 (170)
En bloc resection	38.5 (129)	37.1 (13)	38.9 (116)
Amputation	3.9 (13)	2.9 (1)	4.0 (12)
**Adjuvant**			
None	16.2 (31)	0.0 (0)	18.2 (31)
Simple	40.8 (78)	28.6 (6)	42.4 (72)
Combined (>2)	42.9 (82)	71.4 (15)	39.4 (67)
**Types of adjuvants**			
Extensive curettage	49.2 (94)	76.2 (16)	45.9 (78)
Phenol/alcohol	18.8 (36)	23.8 (5)	18.2 (31)
Fulguration	66.0 (126)	76.2 (16)	64.7 (110)
**Filling type**			
Cement	95.8 (183)	90.5 (19)	96.4 (164)
Bone graft	1.0 (2)	0.0 (0)	1.2 (2)
No filling	3.1 (6)	9.5 (2)	2.4 (4)
Denosumab	9.5 (32)	0.0 (0)	10.8 (32)

Categorical variables are presented as frequencies and percentages.

### Local recurrence rates

The overall local recurrence rate was 15.8% (53/335). Curettage led to a 21.4% recurrence rate, whereas *en bloc* resection resulted in a 9.3% recurrence rate. There was a 25% recurrence rate among patients with a single surgical adjuvant and 14% among those with multiple adjuvants, compared to 29% with no adjuvants. Post-curettage recurrence was least common in the proximal tibia at 19%, followed by the distal femur at 22.5%, and 40% in the proximal fibula. *En bloc* resection recurrence rates were lower in all three anatomical locations. No recurrences were reported post-amputation. Curettage combined with denosumab indicated a slight increase in recurrence (23.8%) compared to curettage alone (21%). The overall recurrence rate decreased from 22.9% in the period 1989-2005 to 15.1% in 2006-2021. While the recurrence rates for curettage remained relatively stable at 23.8% for 1989-2005 and 21.2% for 2006-2021, there was a notable reduction in recurrence after *en bloc* resection, dropping from 23% to 7.8% over the same periods (Table 3).

**Table 3:** Overall and overtime (1989-2005 and 2006-2021) local recurrence rates.

Local recurrence %(n)	All patients	1989-2005	2006-2021
	(n=335)	(n=35)	(n=300)
Overall	15.8% (53/335)	22.9% (8/35)	15.1% (45/300)
Curettage	21.4% (41/191)	23.8% (5/21)	21.2% (36/170)
En bloc	9.3% (12/129)	23.1% (3/13)	7.8% (9/116)
Amputation	0/13	0/1	0/12
Curettage after denosumab	23.8% (5/21)	0	23.8% (5/21)

Categorical variables are presented as frequencies and percentages.

## DISCUSSION

In this study we report on 335 GCTB cases of the knee treated in Brazil over a 30-year period, featuring insights into demographic distribution, tumor localization, and aggressiveness, as well as treatment strategies and their corresponding outcomes. The overall local recurrence rate was 15.8%, with a discernible higher risk associated with curettage (21%) compared to *en bloc* resection (9%). Notably, despite variations in adjuvant therapy use, recurrence rates post-curettage remained significantly high, particularly emphasizing the concern for patients without surgical adjuvant therapy (29%). A time-based analysis showed a reduction in the overall local recurrence rate. While recurrence rates after curettage remained consistent across the study periods, there was a considerable decrease in recurrences after *en bloc* resections, dropping from 23% to 7.8%. This decline is likely attributed to the use of MRI for preoperative planning. Furthermore, the period comparison underscored an increased presence of more aggressive tumors in recent years, with Campanacci grade 3 cases rising from 43% to 58%. In relation to the use of denosumab prior to curettage, cases that received denosumab exhibited a slight increase in the rate of local recurrence compared to those undergoing isolated curettage. Recently, do Brito et al.^5^ published a systematic review encompassing studies from 2005 to 2019, highlighting that curettage, particularly when supplemented with adjuvants, typically yields acceptable local recurrence rates, often under 15%. This contrasts the higher rates (20-50%) often reported in earlier literature.^10,11^ We report a local recurrence rate of 21% post-curettage, with a substantial drop to 14% with the integration of multiple adjuvants. These adjuvants also generally included extended tumor removal with a high-speed burr (extended curettage). Similarly, Niu et al.^12^ reviewed 283 patients with GCTB (60% in the knee region), reporting a local recurrence of 12.4%, with 8.6% for extended curettage and 56.1% for curettage alone. Capanna et al.^13^ supported these findings, showing a 16% local recurrence when adjuvants were used, versus 37% for standalone curettage. This suggests that variations in local recurrence are evident across different studies, and adjuvants appear to aid in reducing the recurrence rate of GCTB.

In 2016, Lin et al.^14^ reported on a series from a multicenter nationwide GCTB registry, encompassing 268 patients treated for GCTB around the knee, with an overall recurrence rate of 21.4% and a high incidence of Campanacci grade 3 tumors at 44%. Similarly, our study reflected comparable trends in local recurrence, yet it highlighted an even more pronounced prevalence of Campanacci grade 3 tumors, accounting for 56.7% of cases. The elevated incidence of Campanacci grade 3 tumors, typically anticipated to be around 20%, could be associated with deferred medical care in developing nations such as Brazil.^15^

It is important to emphasize the potential ramifications of delayed treatment, underlining the impact of timely clinical evaluations and interventions in the management of GCTB. Errani et al.^16^ in their study of 349 patients with GCTB highlighted that more aggressive tumors, particularly those graded as Campanacci 3, correlated with *en bloc* resections and a heightened risk of lung metastasis. Similarly, Medellin et al.^17^ reported higher rates of pathological fractures in Campanacci 3 tumors compared to Campanacci 2 tumors among a cohort of 107 patients. While a meta-analysis by Salunke et al.^18^ indicated that the presence of a fracture did not increase the risk of local recurrence in GCTB cases, other studies^6,19,20^ have documented an elevated recurrence rate in stage 3 tumors.

Building on this, our research reaffirms the necessity of meticulous tumor management strategies to minimize GCTB recurrences. Our approach endorses the systematic application of adjuvants during curettage and meticulous planning for achieving optimal margins in *en bloc* resections. Recognizing the implications, we also advocate for enhancing healthcare policies to facilitate patient access to medical services. This strategy is crucial for musculoskeletal malignancies, which mirror the challenges seen in GCTB, often marked by late presentations and high-risk patients. One approach would be the establishment of a national database and the standardization of clinical protocols and best practices.

An inherent limitation of our GCTB study is its retrospective nature, which can introduce potential systematic biases. This is a common challenge in retrospective research, where the risk of selection bias, information bias, and confounding variables are heightened. It is crucial to approach our findings with caution, recognizing that while they offer valuable insights, they are based on historical data from 30-year time period, and there might be factors not accounted for that could influence the outcomes.^21^

## CONCLUSIONS

In summary, we report on a large multicenter cohort of GCTB of the knee in Brazil over a 30-year period. There was a high incidence of Campanacci grade 3 tumors, particularly in the more recent 15-year time period; however, the overall local recurrence rate of 15.8% is consistent with previous literature. Over time, the overall local recurrence rate declined, particularly following *en bloc* resection. Our findings highlight the challenges of treating rare diseases in the context of an emerging economy.

## References

[1] Mavrogenis AF, Igoumenou VG, Megaloikonomos PD, Panagopoulos GN, Papagelopoulos PJ, Soucacos PN (2017). Giant cell tumor of bone revisited. SICOT J.

[2] Palmerini E, Picci P, Reichardt P, Downey G (2019). Malignancy in Giant Cell Tumor of Bone: A Review of the Literature. Technol Cancer Res Treat.

[3] van der Heijden L, Dijkstra S, van de Sande M, Gelderblom H (2020). Current concepts in the treatment of giant cell tumour of bone. Curr Opin Oncol.

[4] World Health Organization (2020). WHO Classification of Tumours.

[5] Soares do Brito J, Spranger A, Almeida P, Portela J, Barrientos-Ruiz I (2021). Giant cell tumour of bone around the knee: a systematic review of the functional and oncological outcomes. EFORT Open Rev.

[6] O'Donnell RJ, Springfield DS, Motwani HK, Ready JE, Gebhardt MC, Mankin HJ (1994). Recurrence of giant-cell tumors of the long bones after curettage and packing with cement. J Bone Joint Surg Am.

[7] Wang J, Liu X, Yang Y, Yang R, Tang X, Yan T (2021). Pulmonary metastasis of giant cell tumour: a retrospective study of three hundred and ten cases. Int Orthop.

[8] Kafchitsas K, Habermann B, Proschek D, Kurth A, Eberhardt C (2010). Functional results after giant cell tumor operation near knee joint and the cement radiolucent zone as indicator of recurrence. Anticancer Res.

[9] Campanacci M, Baldini N, Boriani S, Sudanese A (1987). Giant-cell tumor of bone. J Bone Joint Surg Am.

[10] Campanacci M, Giunti A, Olmi R (1975). Giant-cell tumours of bone: a study of 209 cases with long term follow up in 130. Ital J Orthop Traumatol.

[11] Goldenberg RR, Campbell CJ, Bonfiglio M (1970). Giant-cell tumor of bone. An analysis of two hundred and eighteen cases. J Bone Joint Surg Am.

[12] Niu X, Zhang Q, Hao L, Ding Y, Li Y, Xu H (2012). Giant cell tumor of the extremity: retrospective analysis of 621 Chinese patients from one institution. J Bone Joint Surg Am.

[13] Capanna R, Fabbri N, Bettelli G (1990). Curettage of giant cell tumor of bone. The effect of surgical technique and adjuvants on local recurrence rate. Chir Organi Mov.

[14] Lin F, Hu Y, Zhao L, Zhang H, Yu Xi, Wang Z (2016). The epidemiological and clinical features of primary giant cell tumor around the knee: A report from the multicenter retrospective study in china. J Bone Oncol.

[15] de Souza PRB, Szwarcwald CL, Damacena GN, Stopa SR, Vieira MLFP, de Almeida WS (2021). Health insurance coverage in Brazil: analyzing data from the National Health Survey, 2013 and 2019. Cien Saude Colet.

[16] Errani C, Ruggieri P, Asenzio MAN, Toscano A, Colangeli S, Rimondi E (2010). Giant cell tumor of the extremity: A review of 349 cases from a single institution. Cancer Treat Rev.

[17] Medellin MR, Fujiwara T, Tillman RM, Jeys LM, Gregory J, Stevenson JD (2018). Prognostic factors for local recurrence in extremity-located giant cell tumours of bone with pathological fracture. Bone Joint J.

[18] Salunke AA, Chen Y, Chen X, Tan JH, Singh G, Tai BC (2015). Does pathological fracture affect the rate of local recurrence in patients with a giant cell tumour of bone?: a meta-analysis. Bone Joint J.

[19] Prosser GH, Baloch KG, Tillman RM, Carter SR, Grimer RJ (2005). Does curettage without adjuvant therapy provide low recurrence rates in giant-cell tumors of bone?. Clin Orthop Relat Res.

[20] Rooney RJ, Asirvatham R, Lifeso RM, Ali MA, Parikh S (1993). Giant cell tumour of bone. A surgical approach to grade III tumours. Int Orthop.

[21] alari K, Goyal M (2020). Retrospective studies - utility and caveats. J R Coll Physicians Edinb.

